# Mediterranean-DASH Intervention for Neurodegenerative Delay (MIND) diet in relation to age-associated poor muscle strength; a cross-sectional study from the Kurdish cohort study

**DOI:** 10.1038/s41598-022-16120-7

**Published:** 2022-07-13

**Authors:** Yahya Pasdar, Shima Moradi, Saman Saedi, Mehdi Moradinazar, Negin Rahmani, Behrooz Hamzeh, Farid Najafi

**Affiliations:** 1 Department of Nutritional Sciences, Research Center for Environmental Determinants of Health (RCEDH), Health Institute, Kermanshah University of Medical Sciences, Kermanshah, Iran; 2 Department of Animal Science, College of Agriculture, Shiraz University, Shiraz, Iran; 3 Behavioral Disease Research Center, Kermanshah University of Medical Sciences, Kermanshah, Iran; 4 Julius Maximillian University of Wuerzburg, Wuerzburg, Germany; 5 Environmental Determinates of Health Research Center, School of Public Health, Kermanshah University of Medical Sciences, Kermanshah, Iran; 6 School of Public Health, Communing Developmental and Health Promotion Research Center, Kermanshah University of Medical Sciences, Kermanshah, Iran

**Keywords:** Health care, Ageing, Metabolism

## Abstract

The Mediterranean-DASH Intervention for Neurodegenerative Delay (MIND) diet is an eating pattern associated with multiple health benefits, including the conservation of skeletal muscle. The Hand Grip Strength (HGS) is the most frequently used indicator of muscle functional capacity and muscle strength for clinical purposes. The current study aims to investigate the association between adherence to MIND diet and prevention of age-associated decline in muscle strength among the Kurdish population in Iran**.** This cross-sectional study was performed using data from Ravansar non-communicable diseases (RaNCD) cohort study on 3181 adults (48.5% men) aged 35–65 years. The dietary intake of the studied participants was assessed using a 114-item food frequency questionnaire (FFQ) developed by RaNCD cohort study. The MIND diet and the major dietary patterns were identified based on the participants’ dietary intake and three dietary patterns emerged including plant-based diet, high protein diet, and unhealthy diet. Hand grip strength (HGS) was measured using a hand-held hydraulic handgrip dynamometer and poor HGS was defined as HGS less than 32.8 and 20.5 kg in men and women, respectively. Compared with participants in the lowest category of MIND diet, those in the highest category had lower odds of poor HGS (OR: 0.65; CI 95%: 0.51–0.83). Furthermore, participants who were in third tertiles of plant-based and high protein diet were more likely 37% and 33% lower odds ratio of poor HGS (OR: 0.63; CI 95%: 0.5–0.79), (OR: 0.67; CI 95%: 0.54–0.84), respectively. On the other hand, greater adherence to the unhealthy diet was increased odds of poor HGS (OR: 1.39; CI 95%: 1.11–1.74). Overall, our findings suggest that adherence to the MIND diet and high protein diet may be associated with higher HGS, while adherence to the unhealthy diet can increase the odds of age-associated poor HGS in the Kurdish population.

## Introduction

Maintaining muscle strength is important for reducing functional limitations. Poor muscle strength represents an important public health problem^1^ and lead to disability and functional impairment^2,3^. Hand Grip Strength (HGS) is the most frequently used indicator of muscle strength and muscle functional capacity for clinical purposes^4^. HGS indicator to identify older adults who are at risk for poor physical function, weakness, and low muscle mass has also been recommended^5^.

The association between nutritional status and HGS is well documented such that HGS is considered a non-invasive and reliable method for assessing nutritional status^6^. The Academy of Nutrition and Dietetics and the American Society for Parenteral and Enteral Nutrition has recently recommended that the HGS is one of the six characteristics to diagnose adult malnutrition^7^. Therefore, adherence to a healthy diet can strengthen muscle strength. However, recent dietary research has focused on the dietary pattern because dietary components are consumed in combination and interact with one another^8,9^.

The Mediterranean-DASH Intervention for Neurodegenerative Delay (MIND) diet has been recently suggested to emphasize foods that impact brain health^10^. The MIND diet is a plant-based, antioxidant-rich diet, and focuses on limiting the intake of animal products and foods high in saturated fat^10^. Components of the MIND diet include vegetables and especially green leafy vegetables, fruits, tree nuts, legumes, berries, whole grains, olive oil, beans, and a moderate intake of fish and poultry^10^. The MIND diet could improve neuronal physiology and anatomy^11^ and the protective association of the MIND diet with the brain and cognitive health has been demonstrated in previous studies^10,12,13^. It has been shown that protection of skeletal muscle strength and physical function is associated with cognitive health^14^. However, limited data are available on examining the adherence to the MIND diet in relation to muscle strength and sarcopenia.

Since the screening of muscle status and nutritional quality of the community is important to prevent non-communicable diseases (NCDs) and considering that the impact of the MIND diet on other aspects of aging such as cognitive health has been reported and it has been shown that muscle strength is associated with cognitive health, we hypothesized that adherence to the MIND dietary pattern would be protective for age-associated poor muscle strength. Therefore, the present study aimed to investigate the association between adherence to MIND dietary pattern and the prevention of age-associated decline in muscle strength among the Kurdish population. For this purpose, we examined the association of MIND diet adherence with muscle strength assessed by HGS, which is a simple, reliable, and non-invasive method applied in many epidemiological studies to measure muscle strength.

In addition, since foods are a set of nutrients that must interact with each other, it is often impossible to differentiate the effects of specific foods^15^. Accordingly, the understanding of a community's dietary patterns has evolved to assess the effects of overall dietary intake. Evaluating dietary patterns increases the ability to assess more fundamental effects due to the cumulative effects of many dietary factors and allows the interaction between synergistic components to be evaluated^16–18^. Therefore, in the present study, in addition to determining and evaluating the MIND diet, the major dietary patterns of the population under study were determined and their relationship with muscle strength was investigated to determine if these patterns are in line with the MIND diet.

## Methods

### Study design and population

The study population of this cross-sectional study was extracted from the baseline phase data from the Ravansar non-communicable diseases (RaNCD) cohort study. Since 2014, this study is the first Kurdish population-based study on 10,047 Kurdish participants aged 35–65 years (4764 men and 5283 women) living in Ravansar city, Kermanshah province, Western Iran. RaNCD is a branch of PERSIAN (Prospective Epidemiological Research Studies in Iran) mega-cohort study which was developed to evaluate non-communicable diseases. The protocol of the RaNCD cohort study was described in the previous studies^19,20^. The ethical approval has been granted by the Ethics Committee of Kermanshah University of Medical Sciences (ethics approval number: KUMS.REC.1394.318).

### Participants

**Figure 1:**
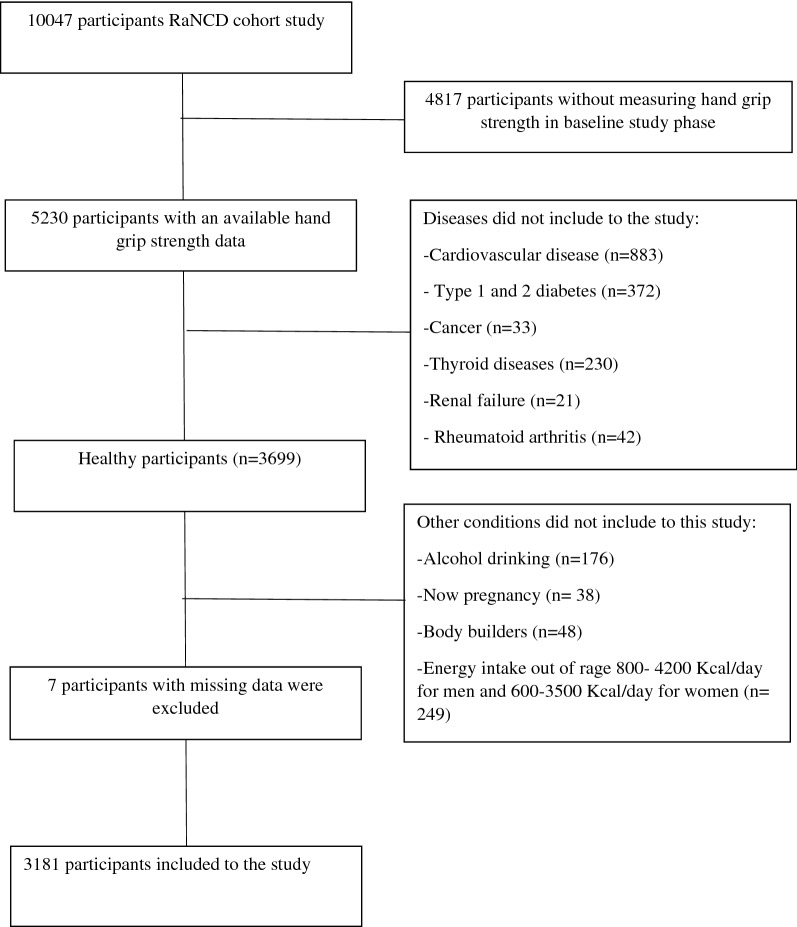
Flow chart of participants selection for this study.

After excluding 4817 participants due to the lack of their muscle strength measurement in the baseline study phase of the RaNCD cohort study, participants with cardiovascular diseases (CVDs), diabetes, thyroid diseases, cancer, renal failure, and rheumatoid arthritis^21–24^ were not included in this study. Pregnant women, alcohol consumers, and body builder participants we also excluded from this study. In the current study, we included only healthy participants since CVD, diabetes, thyroid diseases, pregnancy, etc. have significant effects on muscle strength. In addition, these conditions seem to lead to changes in diet and/or specific medication use. Moreover, due to the possibility of under- or over-reporting of dietary intake, individuals with a total energy intake outside the range of 800–4200 kcal/day for men and 600–3500 kcal/day for women were excluded (n = 249). Seven participants with missing data were excluded from the study. Ultimately, 3181 participants took part in the study. (Fig. 1).

### Data sources/measurements

This study was developed by the PERSIAN (Prospective Epidemiological Research Studies in Iran) mega cohort study and was approved by the Ethics Committees in the Ministry of Health and Medical Education, the Digestive Diseases Research Institute, Tehran University of Medical Sciences, Iran. The details of this study were taken from previous studies. The main phase of the study started in March 2015 for the purpose of identifying dietary and lifestyle conditions related to several diseases and disorders. Overall, 15,000 people aged 35–65 years were living in both urban and rural areas of Ravansar district. The investigators decided to include 10,000 people based on all available resources and in agreement with the central PERSIAN team. To increase the feasibility of the study, people were recruited from both urban and rural areas. The size of the sample recruited for the study was proportional to the total population covered by each health center. By the end of February 2017, 10,047 participants had been recruited, including the 1100 from the pilot phase (from which data have been collected using the revised questionnaires and procedures). All the study staff, including a medical doctor, interviewers, laboratory technicians, executive managers, and receptionists were selected from local applicants by face-to-face interviews with principal investigators. The basic data including demographics, dietary intake, physical activity (PA), smoking status (never, current, or former smoker), dynamometry, anthropometric indices, and body composition were obtained by the study staff. Current and past medical history, family history, and medication use were assessed by the RaNCD physician^20^.

### Physical activity

The standard questionnaire designed by PERSIAN mega cohort study was used to assess physical activity in which 22 questions concerned the level of daily activity. After collecting the data, the participants’ responses were reported based on the metabolic equivalent of task per hour per day (MET/h/day). Details of this questionnaire were published in the previous study^19^.

### Anthropometric indices

InBody 770 device (Inbody Co, Seoul, Korea) was used to measure weight and body composition including body fat mass (BFM), free fat mass (FFM), percentage body fat (PBF), soft lean mass (SLM), and skeletal muscle mass (SMM) while participants were minimally clothed. The height of RaNCD participants was measured using the automatic stadiometer BSM 370 (Biospace Co., Seoul, Korea) in a standing position without shoes with a precision of 0.1 cm. Body mass index (BMI) was computed using this formula: weight (kg) divided by height squared (m^2^). Furthermore, waist circumference (WC) was measured by a non-stretched and flexible tape in the standing position at the level of the iliac crest by a trained nutritionist.

### Hand grip strength

Hand grip strength (HGS) was evaluated with the dominant hand using a hand-held calibrated hydraulic hand grip dynamometer (model SH5003; Saehan Corporation, Masan, Korea). To measure HGS, participants were asked to sit on a chair and flex their elbows at a 90° to the arm of the chair handle. Then participants were asked to press the dynamometer handle with maximum power for 10 s. After 30 s, the measurement was repeated, and the average values ​​recorded by the dynamometer were reported as the individual's final HGS. Calibration and validation of this device was published in the previous study^25^. We considered the cut-off point reported by Lauretani et al.^26^ to assess age related poor muscle strength.

### Dietary intake assessment

A validated semi-quantitative 118-item food frequency questionnaire (FFQ) was applied to evaluate the dietary intake of RaNCD participants^27,28^. As for nutrients, these 118 food items were broken down into thirty one food groups (principal components)^29^. The major dietary patterns were extracted by principal component analysis (PCA) and factors were rotated using the varimax method to minimize the number of variables. As a result, the first three major dietary patterns with eigenvalues more than 1.5 were extracted based on the screen diagram and the interpretability of the factors. The factor loading is the degree of correlation between food groups with each dietary pattern. We explained and named the dietary patterns considering a factor load more than 0.3. Finally, for each participant, the factor load for each dietary pattern was calculated. Factor loading indicates the extent to which each participant adheres to a known dietary pattern. A higher and more positive factor loading for a dietary pattern means more adherence to this pattern, while a low factor loading score indicates a lower adherence to the dietary pattern.

### MIND diet

To determine the MIND diet, we used the data derived from RaNCD cohort FFQ according to the method that was first proposed by Morris et al.^10^. The MIND diet components that we used in this study consisted of two parts: (a) 10 healthy food groups for the brain (green leafy vegetables, other vegetables, berries, nuts, whole grains, fish, beans, poultry, olive oil and wine); and (b) 5 unhealthy food groups for the brain (butter and margarine, cheese, red meat and meat product, fast fried foods, pastries and sweets). Each of the mentioned food groups was divided into three categories. To score the MIND diet, participants who consumed the healthiest foods for the brain in the lowest tertile were given a score of 0. A score of 0.5 was given to the middle tertile of these food groups and those in the third tertile were given a score of 1. In the case of unhealthy brain food groups, the scoring was reversed. In this way, the participants who received unhealthy foods in the highest tertile were given a score of zero. In the present study, due to a lack of information on alcohol consumption, the related data were not included in the main data set. Overall, the scores of each component were summed up and the final score of MIND diet was reported between 0 and 14.

### Statistical analysis

Basic characteristics of participants including quantitative variables were reported by mean ± standard deviation (SD), and qualitative variables were presented using frequency (%). These characteristics were compared based on the tertiles of MIND diet using one-way ANOVA and Chi-square tests. Multiple-crude and adjusted odds ratios (OR) and 95% confidence intervals (CI 95%) were applied to evaluate the odds ratio of age-associated poor muscle strength in participants with the MIND diet and other dietary patterns.

Potential confounders were adjusted for in our analyses. They were adjusted for age (continuous) and gender (categorical) in model 1. In model 2, adjustment was done for variables in model 1 as well as for physical activity (continuous), BMI (continuous), and smoking (categorical). In model 3, adjustment was done for all mentioned variables in previous models plus energy intake (continuous) and treatment (categorical). Radar graph was drawn to better demonstrate the relationship between the components of MIND diet and HGS.

In this study, there was no normal distribution for the HGS variable and the data had positive skewness. On the other hand, in the study by Lauretani colleagues^26^, sarcopenia was considered based on the muscle strength cut-off point. In addition, the effect of the major dietary patterns and MIND diet on muscle strength was not constant with increasing units. Therefore, in this study, according to the appropriate interpretation, the logistic regression models were used.

All statistical analyses were performed using SPSS 20 (IBM Corp, Chicago, IL, USA). *P* values were considered significant at the level of < 0.05.

### Ethical approval

The Ethics Committee of Kermanshah University of Medical Sciences approved the study (code: KUMS.REC.1392.318). All methods were carried out in accordance with relevant guidelines and regulations. All the participants were provided oral and written informed consent.

### Informed consent

Written informed consent was obtained from each studied subject after explaining the purpose of the study. The right of subjects to withdraw from the study at any time and subject's information is reserved and will not be published.

## Results

A total of 3181 adults (48.5% men) were included in this cross-sectional study. Mean of SLM, FFM, and SMM significantly increased with higher adherence to the MIND diet. In addition, HGS in the third tertile was significantly higher than the first tertile (*P* < 0.001). There was no significant difference in the mean physical activity in the tertiles of the MIND diet. Other baseline participant characteristics are reported in Table 1.

**Table 1:** Basic characteristics of participants.

Characteristics	Total (n = 3181)	Tertiles of MIND Diet			*P* value**
		T1 (n = 1309) (< 6)	T2 (n = 826) (6–7.5)	T3 (n = 1046) (7.5–12.5)	
Age (year)	46.17 ± 8*	46.81 ± 8.35	45.93 ± 7.94	45.45 ± 7.36	< 0.001
Gender, male (%)	48.5	37	52	63.1	< 0.001
Weight (kg)	70.7 ± 13.71	68.68 ± 13.45	70.90 ± 13.44	73.90 ± 13.94	< 0.001
BMI (kg/m^2^)	26.62 ± 4.63	26.47 ± 4.67	26.56 ± 4.62	26.95 ± 4.56	0.072
WC	96.83 ± 10.38	96.41 ± 10.57	96.59 ± 10.24	97.93 ± 10.17	0.004
BFM (kg)	23.47 ± 9.29	23.45 ± 9.32	23.29 ± 9.35	23.77 ± 9.16	0.542
SLM (kg)	44.69 ± 8.95	42.82 ± 8.68	45.01 ± 8.65	47.43 ± 9.09	< 0.001
FFM (kg)	47.24 ± 9.44	45.27 ± 9.14	47.58 ± 9.14	50.14 ± 9.60	< 0.001
SMM (kg)	26.13 ± 5.72	24.93 ± 5.54	26.34 ± 5.54	27.90 ± 5.81	< 0.001
PBF (%)	32.57 ± 9.41	33.48 ± 9.46	32.20 ± 9.46	31.59 ± 9.10	< 0.001
HGS (kg)	32.61 ± 11.31	30.12 ± 10.52	33.21 ± 11.36	36.01 ± 11.54	< 0.001
PA (MET hour/day)	41.67 ± 7.77	41.89 ± 7.09	41.58 ± 8.33	41.44 ± 8.04	0.398
Smoking (%)	18.4	16	18.7	22.1	0.003
Obese (%)	20.8	20.5	19.1	22.6	0.472
Hypertension (%)	4	3.5	3.9	4.8	0.27
Hypercholesterolemia (%)	16.9	16.5	18.2	16.4	0.527

*Mean ± SD.

**P values were obtained one-way ANOVA and Chi square.

*BMI* body mass index, *WC* waist circumference, *BFM* body fat mass, *SLM* soft lean mass, *FFM* free fat mass, *SMM* skeletal muscle mass, *PBF* percentage body fat, *HGS* hand grip strength, *PA* physical activity.

Three major dietary patterns were extracted using PCA including: (1) plant- based diet that was high in leafy vegetables, fresh fruits, starchy vegetables, dried fruits, potato, nuts, tomato, carotene-rich vegetables, etc.; (2) high protein diet dominated by red meat, viscera, processed meat, fish, poultry, and legumes; and (3) unhealthy diet with high loadings of hydrogenated fats, sweets and desserts, tea and coffee, salt, refined grain, and low loading for fruits, vegetable, vegetable oil and olive (Table 2).

**Table 2:** Factor loading of food groups in the major identified dietary patterns.

Food groups	Major dietary patterns		
	Plant- based dietary pattern	High protein dietary pattern	Unhealthy dietary pattern
Leafy vegetables	.654		
Fresh fruits	.600	.259	− .215
Starchy vegetables	.568		
Dried fruits	.469		− .250
Potato	.452		.270
Nuts	.448	.364	
Tomato	.422		.213
Carotene-rich vegetables	.420	.213	− .375
Condiments	.397		
Dairy	.396		
Butters	.378		
Pickles	.327		
Egg	.314	.239	
Poultry	.285	.241	
Whole grains	.236	.224	
Red meat		.672	
Viscera		.621	
Fish		.617	
Soft drinks		.462	.244
Processed meat		.387	
Legumes	.333	.378	
Natural juices	.230	.289	
snack		.240	
Hydrogenated fats			.587
Sweets and desserts	.244	.225	.549
Tea and coffee			.538
Vegetable oil	.243		− .403
Olive		.216	− .328
Salt			.317
Refined grains		.258	.278
Variance %	11.04	8.48	6.73

Factor loading less than 0.2 have been removed for clarity.

Table 3 provides the association between adherence to the MIND diet and other major dietary patterns with HGS. We found that participants who were in the third tertile of MIND diet had a 43% a lower odds ratio of poor HGS (OR: 0.57; CI 95%: 0.45–0.71). In addition, after controlling for potential confounders including age, gender, physical activity, BMI, and smoking, this diet had a significant preventive association with poor HGS (OR: 0.65; CI 95%: 0.51–0.83). Similarly, compared with those in the lowest tertile, those in the highest tertile of plant-based and high protein dietary patterns were 47% and 49% less likely to be poor HGS either before (OR: 0.53; CI 95%: 0.43–0.66 and OR: 0.51; CI 95%: 0.41–0.63, respectively) or after controlling for potential confounders (OR: 0.63; CI 95%: 0.5–0.79 and OR: 0.67; CI 95%: 0.54–0.84, respectively). In addition, participants with greater adherence to the unhealthy dietary pattern were 1.49 times more likely to be poor HGS (OR: 1.49; CI 95%: 1.21–1.84). This association remained after controlling for the mentioned potential confounders (OR: 1.39; CI 95%: 1.11–1.74) (Table 3).

**Table 3:** Multiple-adjusted odds ratios and 95% confidence intervals for hand grip strength across of the MIND diet and major identified dietary patterns.

Dietary patterns		Odds ratio (95% CI)			P-trend
		T1	T2	T3	
MIND diet	Crude	1	0.82 (0.68–0.99)	0.57 (0.45–0.71)	< 0.001
	Model 1	1	0.89 (0.73–1.08)	0.63 (0.5–0.81)	0.004
	Model 2	1	0.9 (0.73–1.1)	0.65 (0.51–0.83)	0.007
	Model 3	1	1 (0.81–1.2)	0.73 (0.59–0.91)	0.008
Plant- based diet	Crude	1	0.79 (0.65–0.97)	0.53 (0.43–0.66)	< 0.001
	Model 1	1	0.9 (0.73–1.11)	0.61 (0.49–0.77)	< 0.001
	Model 2	1	0.92 (0.74–1.13)	0.63 (0.5–0.79)	< 0.001
	Model 3	1	0.98 (0.78–1.22)	0.72 (0.55–0.94)	0.02
High protein diet	Crude	1	0.75 (0.62–0.92)	0.51 (0.41–0.63)	< 0.001
	Model 1	1	0.88 (0.71–1.08)	0.66 (0.53–0.83)	< 0.001
	Model 2	1	0.88 (0.71–1.09)	0.67 (0.54–0.84)	0.001
	Model 3	1	0.93 (0.75–1.16)	0.77 (0.6–0.99)	0.046
Unhealthy diet	Crude	1	1.21 (0.98–1.49)	1.49 (1.21–1.84)	< 0.001
	Model 1	1	1.11 (0.88–1.38)	1.33 (1.07–1.66)	0.008
	Model 2	1	1.12 (0.89–1.4)	1.39 (1.11–1.74)	0.003
	Model 3	1	1.19 (0.95–1.5)	1.77 (1.38–2.26)	< 0.001

*Model 1 adjusted for age and gender.

*Model 2 adjusted for variables in model 1, physical activity, BMI, and smoking.

*Model 3 adjusted for all variables in previous models plus energy intake.

We also examined the association between the components of MIND diet and HGS (Table 4). Among them, those who had higher score of green leafy vegetables, other vegetables, berries, whole grains, fish, and olive were less likely to be poor HGS. On the other hand, those who were adhered to unhealthy foods including red meat and its products, fast fried foods, pastries, and sweets had the higher odds ratio of poor HGS (Table 4).

**Table 4:** Multiple-adjusted odds ratios and 95% confidence intervals for hand grip strength across of the components of MIND diet.

Components of MIND diet	OR (CI 95%)*	*P* value
*Brain healthy foods*		
Green leafy vegetables	0.79 (0.62–0.99)	0.048
Other vegetables	0.57 (0.45–0.72)	< 0.001
Berries	0.65 (0.52–0.81)	< 0.001
Nuts	0.95 (0.75–1.2)	0.693
Whole grains	0.77 (0.67–0.97)	0.027
Fish	0.69 (0.55–0.86)	0.001
Beans	0.9 (0.71–1.12)	0.364
Poultry	1.08 (0.88–1.32)	0.427
Olive	0.51 (0.4- 0.65)	< 0.001
*Brain unhealthy foods*		
Butter, margarine	1.06 (0.85–1.33)	0.558
Cheese	0.93 (0.73–1.12)	0.384
Red meat and products	1.43 (1.12–1.83)	0.004
Fast fried foods	1.33 (1.07–1.67)	0.01
Pastries and sweets	1.42 (1.12–1.8)	0.003

*Adjusted for all variables in Table 3.

**Figure 2:**
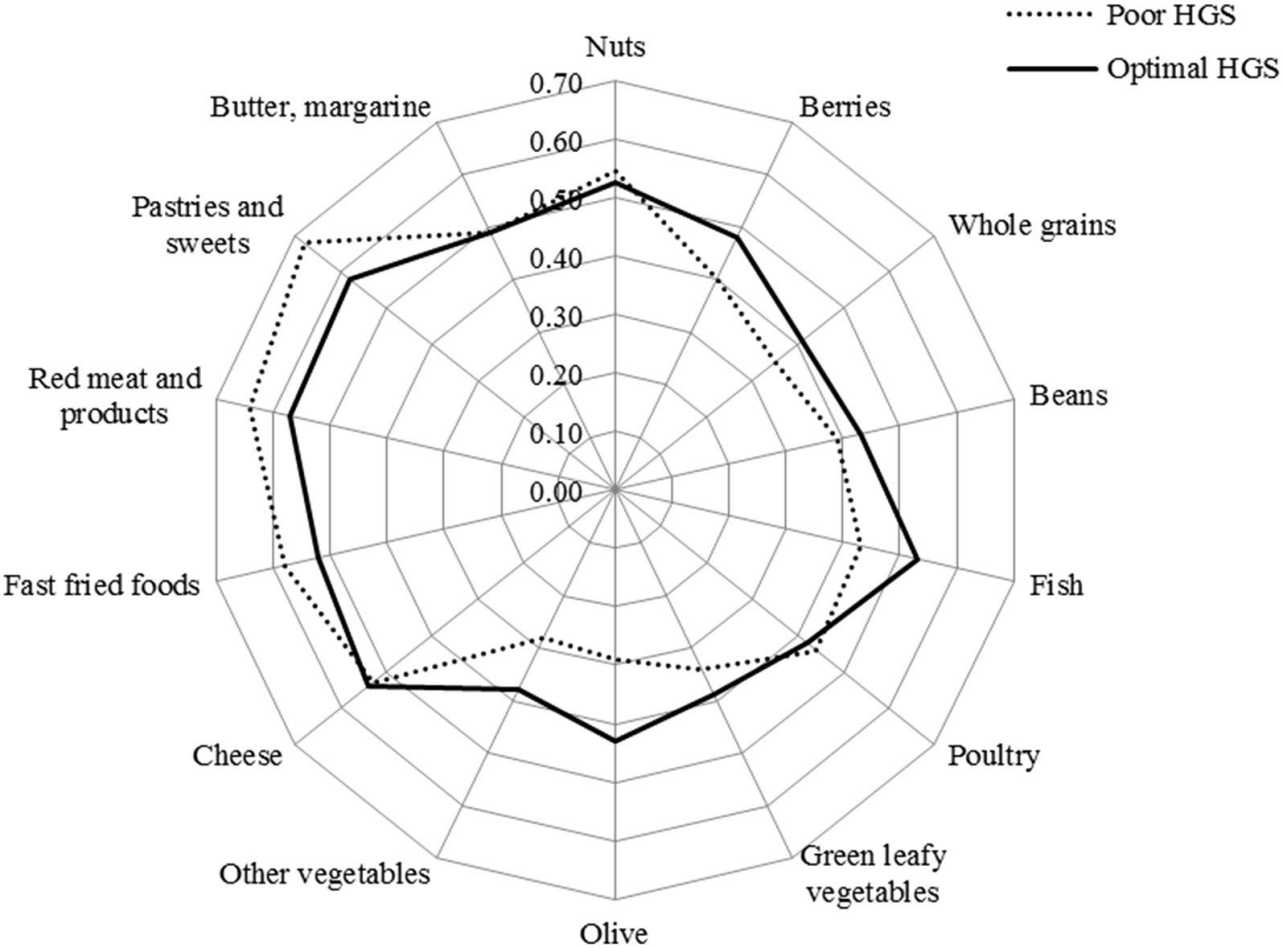
Radar graph for the mean of components of MIND diet in weak and optimal hand grip strength (HGS).

Figure 2 shows radar graph for differences of MIND diet components between participants with poor and optimal HGS.

## Discussion

In this cross-sectional study, we investigated the relationship between adherence to MIND diet patterns and muscle strength. This study was the first on a large sample size to evaluate the relationship between the MIND dietary pattern and age-related poor HGS among the Kurdish population. Our findings showed that adherence to the MIND diet significantly lowered the odds ratio of poor HGS and had protective effects on age-related poor HGS. Such finding was also seen plant based and high protein dietary patterns. Among the components of the MIND diet, higher score of green leafy vegetables, other vegetables, berries, whole grains, fish, and olive were associated with a lower odds ratio of poor HGS. While unhealthy foods for brain including red meat and its products, fast fried foods, pastries, and sweets were associated with the higher odds ratio of poor HGS. Additionally, compared to participants in first tertile, those in the highest tertile of unhealthy dietary pattern had higher odds of poor HGS.

Dietary patterns can play a critical role in maintaining skeletal muscle health and strength^30^. In agreement with our findings, a study has shown a positive association between adherence to the Mediterranean diet (MED) and muscle strength^8^. Furthermore, It has been demonstrated that older Korean men with higher intake of fruits, vegetables, potatoes, whole grains, fish (main components of MIND diet), eggs, seaweed, mushrooms, and legumes had higher muscle mass compared to those with a ‘Western’ dietary pattern^31^. The MIND diet is a mainly plant-based dietary pattern, which has been designed as a combination of Mediterranean and dietary approaches to stop hypertension (DASH) dietary patterns. It has been shown that adherence to the MIND diet has been inversely associated with the risk of cognitive decline, depression, and psychological distress^12,32^. Although the MIND diet was explicitly created for the prevention of cognitive decline, its dietary components may also benefit physical functional health and muscle strength.

In consist with our findings, the present study has been reported a positive significant association between adherence to the MIND diet and HGS^33^. In our study, among the components of the MIND diet, higher adherence to green leafy vegetable berries, whole grains, fish, and olive were associated with a lower odds ratio of poor HGS. Vegetables are rich in inorganic nitrate, and higher dietary nitrate has been associated with better physical function and HGS^34^. Moreover, fish intake has been associated with improved HGS in older men and women^35^, and n–3 fatty acids have demonstrated beneficial effects on muscle mass and strength^36^. Similarly, the higher intake of fruits as a component of the MIND diet had beneficial effects on HGS, muscle strength, as well as FFM^8,37^.

In our study, higher score of green leafy vegetables, other vegetables, berries, whole grains, fish, and olive were significant inversely associated with the odds ratio of age-associated poor HGS. These dietary components are sources of folate, vitamin E, carotenoids, and flavonoids^38^, which have been shown to protect the brain through their anti-inflammatory and antioxidant properties^39,40^, and inhibition of beta-amyloid deposition^41^. In recent years, several studies have discussed the potential role of nutrition, nutritional supplements, and nutrients from foods in skeletal muscle health and age-related muscle loss^42,43^. However, fewer studies have examined the relationships between plant-based diet (e.g., fruits and vegetables, grains, olive)^44,45^ and muscle health in older adults^46,47^. Moreover, the associations between bioactive compounds including curcumin, folate, and antioxidant compounds and muscle ageing are poorly understood^30^. Therefore, these novel dietary candidates that act via mechanisms such as anti-inflammation, anti-oxidation, and anabolic-promoting functions are suggested to prevent age-related muscle loss.

In the present study, plant-based and high protein dietary patterns decreased the odds ratio of poor HGS. Nevertheless, participants with greater adherence to the unhealthy dietary pattern were at greater risk for developing poor HGS. Previous studies have reported an association between high protein intake and skeletal muscle function in the older adults^48,49^ which is in accordance with the results of the current study. Additionally, HGS can be influenced by the intake of specific single nutrients including protein^50^, which may be due to positive associations between muscle protein synthesis and dietary protein intake^51^. High protein intake between 1.0 and 1.2 g/kg/day is appropriate for musculoskeletal health^52^. Our recent findings have demonstrated that adherence to the healthy eating index (HEI)-2015 could promote muscle strength^9^. Among the HEI-2015 components, higher intake of fruit and lower consumption of added sugar had dramatically positive effects on HGS^9^.

Adherence to unhealthy dietary pattern such as butter, margarine, red meat and its products, fast fried foods, pastries, and sweets leads to the accumulation of free radicals and reactive oxygen and nitrogen species (ROS/NRS) by inducing oxidative stress in several organelles in myofibers^30^. Accumulation of ROS/NRS in skeletal muscle leads to impaired cellular homeostasis and damage to key cell macromolecules such as proteins, nucleic acids, and lipids, affecting their structure^53^. A higher intake of beneficial foods in a healthy diet may provide not only adequate energy but also sufficient levels of relevant myo-protective nutrients and bioactive compounds^30^. The action of nutrients acting upon the muscle might help to preserve or improve myofiber quality and quantity by counteracting the loss of muscle strength and pathophysiology of sarcopenia^53–55^. A diet rich in antioxidants such as vitamins, carotenoids, flavonoids, and minerals^43^ from fruits, green leafy vegetables, berries, whole grains olive oil, and nuts such as the MED diet can help in restoring the redox homeostasis^56^ in the muscle and counteract reactive ROS/NRS-induced damage^30^. Therefore, plant-based diets such as MED and MIND diets may provide the appropriate combination of antioxidants in the amounts beneficial to improve homeostasis in myofibers.

## Conclusion

To sum up, the results of the present study support that the MIND diet, plant-based, and high protein dietary patterns are associated with protective effects on age-related poor HGS. Although the role of MIND diet has been recently reported in disease prevention, especially in cognitive decline, the present study demonstrated that this pattern diet could be potentially beneficial for the prevention of age- associated poor HGS and loss of muscle strength.

### Limitations

The current study was the first on a large sample size to evaluate the relationship between the MIND dietary pattern and age-related poor HGS among the Kurdish population. However, this study suffered from some limitations. First, this is a cross-sectional study and the cause-and-effect relationship was not clear. Second, dietary intake was assessed by FFQ, and the error of recalling food intake should not be ignored. However, the questionnaire was presented by trained nutritionists. Nevertheless, our findings need to be confirmed in prospective studies. Therefore, further studies are recommended without these limitations.

## Data Availability

Data will be available upon request from the corresponding author.
